# Relationship Between C-Reactive Protein/Serum Albumin Ratio, Neutrophil/Lymphocyte Ratio, and ANCA-Associated Vasculitis Activity: A Retrospective Single Center Cohort Study

**DOI:** 10.3389/fmed.2022.855869

**Published:** 2022-03-18

**Authors:** Yao Tian, Na Liu, Hui Yin, Lihua Duan

**Affiliations:** ^1^Department of Rheumatology and Clinical Immunology, Jiangxi Provincial People's Hospital, Medical College of Nanchang University, Nanchang, China; ^2^Department of Rheumatology and Clinical Immunology, Jiangxi Provincial People's Hospital, The First Affiliated Hospital of Nanchang Medical College, Nanchang, China

**Keywords:** CAR, NLR, associated vasculitis (ANCA), MPV, disease activity

## Abstract

**Objectives:**

To evaluate the role of C-reactive protein/albumin ratio (CAR), neutrophil/lymphocyte ratio (NLR), and mean platelet volume (MPV) in newly diagnosed AAV patients and examine their clinical significance.

**Methods:**

Data from 79 untreated newly diagnosed AAV patients were collected and 76 health examination subjects were included in the healthy control group. All clinical characteristics of AAV patients were extracted from their medical records. The NLR, CAR, and MPV levels of AAV patients and the healthy controls were compared and the correlation between these markers and clinical characteristics was analyzed. Patients were then divided into two groups based on the 2003 Birmingham Vasculitis Activity Score (BVAS). The correlation between NLR, CAR, and MPV and disease activity was analyzed and their effects on the cumulative survival rate were analyzed.

**Results:**

Compared with the healthy control group, elevated CAR, NLR, and MPV were observed in AAV patients. CAR (*r* = 0.701, *P* < 0.0001) and NLR (*r* = 0.369, *P* < 0.05) were positively correlated with the BVAS while MPV did not show any significant correlation (*P* = 0.85). The optimal cutoff value for disease activity evaluation using CAR was 0.80 (sensitivity: 85% and specificity: 82%, *P* < 0.05). The optimal cutoff value for disease activity evaluation using NLR was 5.15 (sensitivity: 66% and specificity: 72%, *P* < 0.05). Kaplan–Meier survival analysis revealed that the all-cause mortality rate was higher in patients with CAR ≥ 0.8 than in patients with CAR < 0.8 (*P* < 0.05). Patients with low NLR also showed a lower cumulative survival rate (*P* < 0.05).

**Conclusions:**

NLR and CAR can reflect the inflammatory response and disease activity in AAV patients, while MPV is not significantly correlated with disease activity in AAV patients. The all-cause mortality rate was higher in patients with high CAR and NLR than in patients with low CAR and NLR.

## Introduction

Anti-neutrophil cytoplasmic antibody (ANCA)-associated vasculitis (AAV) is a group of rare autoimmune diseases that are characterized by small blood vessel necrosis inflammation and trace or no immune complex deposition in vascular walls. AAV often involves the lungs, ears, nose, throat, kidneys, skin, and nervous system. The pathogenesis is the occurrence of autoantibodies against myeloperoxidase (MPO-ANCA) or proteinase 3 (PR3-ANCA) in AAV patients (1–3). Cardiovascular disease secondary to AAV is the most common cause of death (4). Currently, AAV treatment mainly involves aggressive control of disease progression and activity. Disease activity affects organ function and survival in patients but there is a lack of reliable and convenient inflammatory markers to guide clinical diagnosis. Recently, many studies have reported that many inflammatory markers, such as the monocyte/lymphocyte ratio (MLR) (5), the platelet/lymphocyte ratio (PLR), the neutrophil/lymphocyte ratio (NLR) (6), and the mean plateletcrit, are associated with inflammation and disease activity in many diseases (7). Moreover, a high C-reactive protein (CRP)/albumin (ALB) ratio (CAR) was found to be associated with poor outcome of cancer patients, and this marker could be used as a predictor of poor cancer prognosis (8). To date, many extensive studies have been conducted on the effects and use of NLR, CAR, and mean platelet volume (MPV) in disease progression and prognosis, such as psoriasis, rheumatoid arthritis, systemic lupus erythematosus, Behçet's syndrome, and Sjögren syndrome (7, 9–12). However, there are very few studies on the significance of these markers in AAV. In the present study, we examined the correlation between CAR, NLR, and MPV levels and disease activity in AAV patients. In this retrospective study, we analyzed whether CAR, NLR, MPV, and the BVAS in newly diagnosed patients can reflect AAV activity. We also carried out a survival analysis to analyze the effects of initially high CAR and NLR on the cumulative survival rate. Here, we found that NLR and CAR can reflect the inflammatory response and disease activity in AAV patients, while MPV is not significantly correlated with disease activity in AAV patients. The all-cause mortality rate was higher in patients with high CAR and NLR than in patients with low CAR and NLR. Taken together, these data suggested that the CAR and NLR can serve as potential markers for the AAV disease activity and predictors of survival prognosis.

## Materials and Methods

### Patients

We conducted a retrospective cohort study on newly diagnosed AAV patients in Jiangxi Provincial People's Hospital from January 2011 to April 2021. AAV diagnosis was based on the American College of Rheumatology (ACR) and 2012 Chapel Hill Consensus Conferences Vasculitis nomenclature (1). The 2003 Birmingham Vasculitis Activity Score (BVAS) was used to evaluate AAV activity in 79 patients (13). All patients were treatment-naive and the exclusion criteria were as follows: (1) other comorbid autoimmune diseases; (2) liver disease; (3) hematologic disease; (4) malignancy; (5) severe infection or infectious disease; and (6) absence of complete medical history. Data of 76 subjects who underwent health examinations in Jiangxi Provincial People's Hospital between June 2012 and April 2021 were used as controls. The epidemiological characteristics of healthy controls were matched to patients. The healthy controls were free from autoimmune disease, liver disease, diabetes, hypertension, hematologic disease, malignancies, and other major underlying disease and no blood test abnormality was found. AAV patients were divided into the active group and the non-active group, with BVASs on diagnosis of >15 and ≤15, respectively. All AAV patients from January 2011 to April 2021 were analyzed. The date of diagnosis was taken as the starting point and the date of death as the end point. Patients who survived during this time period were considered as censored data. The patients who were lost to follow-up or did not regularly take medicine were excluded from the survival analysis. Informed consent was obtained from all recruits to this study. This study (No. 2021-06-013) was approved by the Ethics Committee of the Jiangxi Provincial People's Hospital in accordance with the World Medical Association Declaration of Helsinki.

### Clinical and Laboratory Data

All clinical and laboratory data were retrospectively collected after the patients' medical records were screened. The following data were collected: age, gender, weight, blood routine analysis results, CRP levels, hepatic and renal functions, AAV subtype and antibody titer, and BVAS. The 2003 BVAS was used to determine AAV activity. The CAR was calculated by dividing the CRP level (g/dl) by the serum albumin level (g/dl). The NLR was calculated by dividing the neutrophil percentage by the lymphocyte percentage.

### Statistical Analysis

All statistical analyses were conducted using SPSS software (version 21 for windows; IBM Corp., Armonk, NY, USA). The Shapiro–Wilk test was used to determine the normality of variable distribution. If the variable was normally distributed, the *t*-test was used to compare the variables between AAV patients and controls. If the variable was non-normally distributed, the Mann–Whitney U test was used. Continuous data that were normally distributed are expressed as the mean ± standard deviation (SD), continuous data with skewed distribution are expressed as median (inter-quartile range), and categorical data are expressed as percentages. Spearman correlation analysis was conducted to determine the correlation between variables. In addition, receiver operating characteristic (ROC) curve analysis was used to determine the sensitivity and specificity of inflammatory markers in predicting ANCA disease activity. Kaplan–Meier survival analysis was conducted to compare the cumulative survival rates between the two groups. *P* < 0.05 was considered to indicate statistically significance.

## Results

### Basic Characteristics of Study Samples

The mean age of patients was 65 and there were 39 males (49%) and 40 females (51%). The mean age of subjects in the control group was 62 and there were 31 males (41%) and 45 females (59%). There were no statistically significant differences in age and gender between patients and controls (*P* > 0.05) and statistically significant differences were present in the other laboratory markers (*P* < 0.05) (Table 1). Patients with AAV exhibited a variety of systemic impairments, of which the lungs, kidneys, cardiovascular, and nervous systems are more common. More than half of patients with AAV have systemic symptoms of fever and fatigue, as well as lung and kidney damage (Table 2).

**Table 1:** General characteristics of AAV patients and healthy controls.

	**AAV patients**	**Healthy controls**	***P***
	**(*n* = 79)**	**(*n* = 76)**	
Age, year	65 ± 12.17	62 ± 9.56	>0.05
Gender (male), %	39.49%	31.41%	>0.05
White blood cell count, 10^9^/L	8.2 (5.46, 11.38)	5.59 ± 1.27	<0.0001
Red blood cell count, 10^12^/L	3.05 ± 0.90	4.16 ± 0.47	<0.0001
Platelet count, /L	269.33 ± 124.54	202.30 ± 48.45	<0.0001
Mean platelet volume, fl	9.98 ± 1.50	11.04± 1.57	<0.05
Plateletcrit, %	0.53 ± 0.10	0.21 (0.19, 0.24)	<0.05
Neutrophil percentage, %	75.15 ± 9.64	56.03 ± 7.88	<0.0001
Lymphocyte percentage, %	15.40 (11.00, 19.50)	32.59 ± 7.86	<0.0001
CRP, mg/L	23.90 (11.00, 61.60)	2.32 (1.73, 3.02)	<0.0001
CAR	0.80 (0.38, 2.38)	0.05 (0.04, 0.07)	<0.0001
NLR	5.01 (3.55, 7.63)	1.80 (1.40, 2.21)	<0.0001
BVAS	15.65 ± 6.35		

*CAR, C-reactive protein/albumin ratio; NLR, neutrophil/lymphocyte ratio; ANCA, anti-neutrophil cytoplasmic antibody (ANCA); MPO, myeloperoxidase; PR3, proteinase 3; P, perinuclear type; C, cytoplasmic type; BVAS, Birmingham Vasculitis Activity Score*.

*Data are expressed as mean ± standard deviation, as median (inter-quartile range), or as numbers (%)*.

**Table 2:** Baseline clinical menifestation of AAV patients.

**Variables**	**Value (%)**
General	59.49%
Cutaneous	8.86%
Mucous membranes/eyes	6.33%
Ear nose throat	8.86%
Pulmonary	54.43%
Cardiovascular	20.25%
Abdominal	3.80%
Renal	77.22%
Nervous systems	12.66%
GPA	12.66%
MPA	81.01%
EGPA	1.27%
C-ANCA (or PR3 ANCA)	12.66%
P-ANCA (or MPO ANCA)	78.48%
Both ANCAs	2.53%
ANCA negative	6.33%

### CAR, NLR, and MPV Levels Were Elevated in AAV Patients

**Figure 1:**
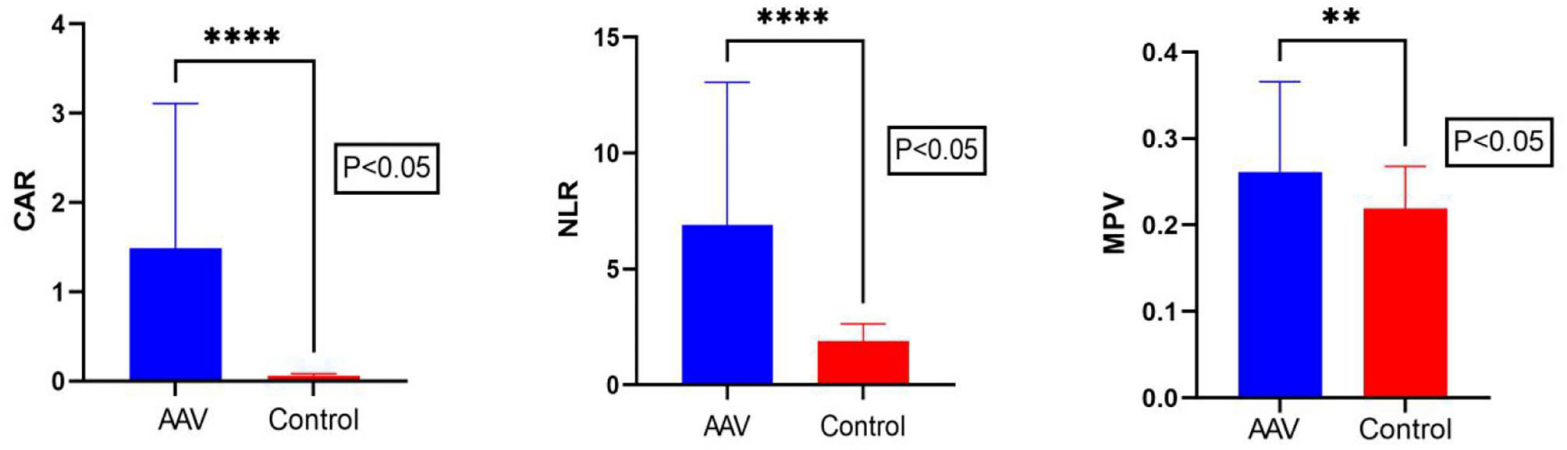
Comparison of CAR, NLR, and MPV levels between AAV patients and the control group. The Mann–Whitney U test showed that the CAR, NLR, and MPV levels in AAV patients were significantly higher than in the control group. CAR, C-reactive protein/albumin ratio; NLR, neutrophil/lymphocyte ratio; MPV, mean platelet volume. The ****symbol indicates *p* < 0.0001 and ** symbol indicates *p* < 0.01.

The CAR of the patient group and the control group was 0.80 (0.38, 2.38) and 0.05 (0.04, 0.07), respectively. The NLR of the patient group and the control group was 5.01 (3.55, 7.63) and 1.80 (1.40, 2.21), respectively. The MPV of the patient group and the control group was 0.53 ± 0.10 and 0.21 (0.19, 0.24), respectively. There were statistically significant differences in NLR? CAR and MPV between the patient group and the control group (*P* < 0.05) (Table 1; Figure 1). We found that the lymphocyte count was lower in AAV patients than in healthy controls (*P* < 0.0001) (Table 1). The CRP level and neutrophil count were significantly elevated in AAV patients (*P* < 0.0001) (Table 1).

### Correlation Between CAR, NLR, and MPV and Clinical Disease Activity in AAV Patients

**Figure 2:**
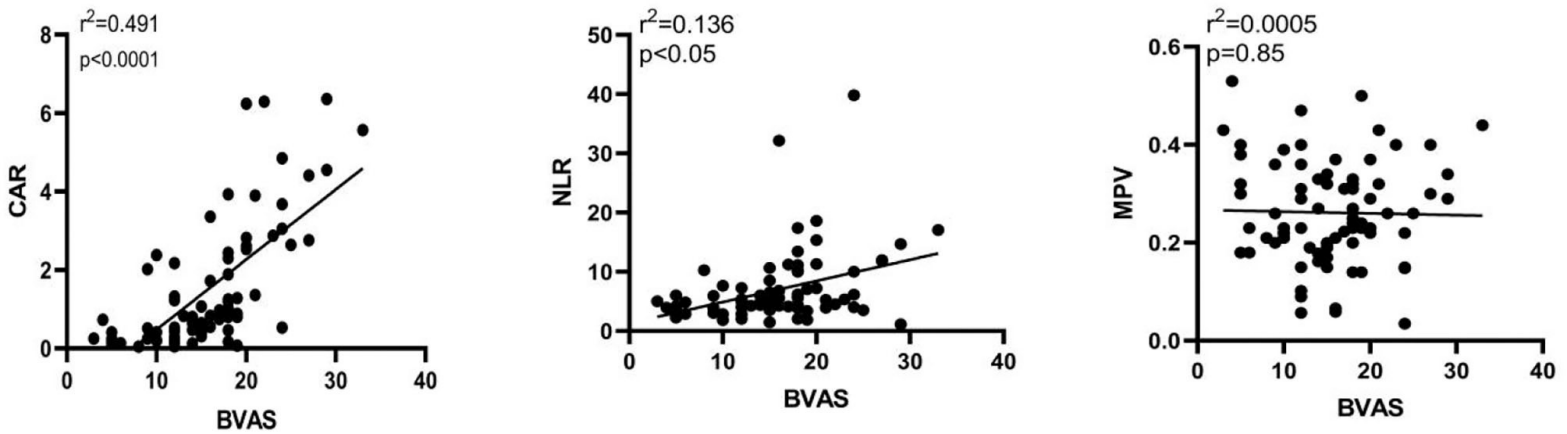
Correlation between CAR, NLR, and MPV levels and disease activity in AAV patients. Spearman correlation analysis was conducted to test the relationship between the CAR, NLR, and MPV and disease activity. The CAR and NLR were found to be positively correlated with disease activity, while MPV did not show a significant correlation with disease activity. CAR, C-reactive protein/albumin ratio; NLR, neutrophil/lymphocyte ratio; MPV, mean platelet volume; BVAS, Birmingham Vasculitis Activity Score.

In our study, the BVAS of AAV patients was positively correlated with CAR (r = 0.701, *P* < 0.0001) and NLR (r = 0.369, *P* < 0.05). The BVAS was not significantly correlated with MPV (*P* = 0.85) (Table 3; Figure 2). A subanalysis between CAR, NLR and MPV based on the presence of MPO or PR3 was performed, while no statistically significant was observed in these analysis, data no shown.

**Table 3:** Correlation between the CAR, NLR, and MPV and BVAS in AAV patients.

	**r**	***P***
CAR	0.701	<0.0001
NLR	0.369	<0.001
MPV	−0.065	>0.05

### ROC Curve Analysis of CAR and NLR for Diagnosing Disease Activity

**Figure 3:**
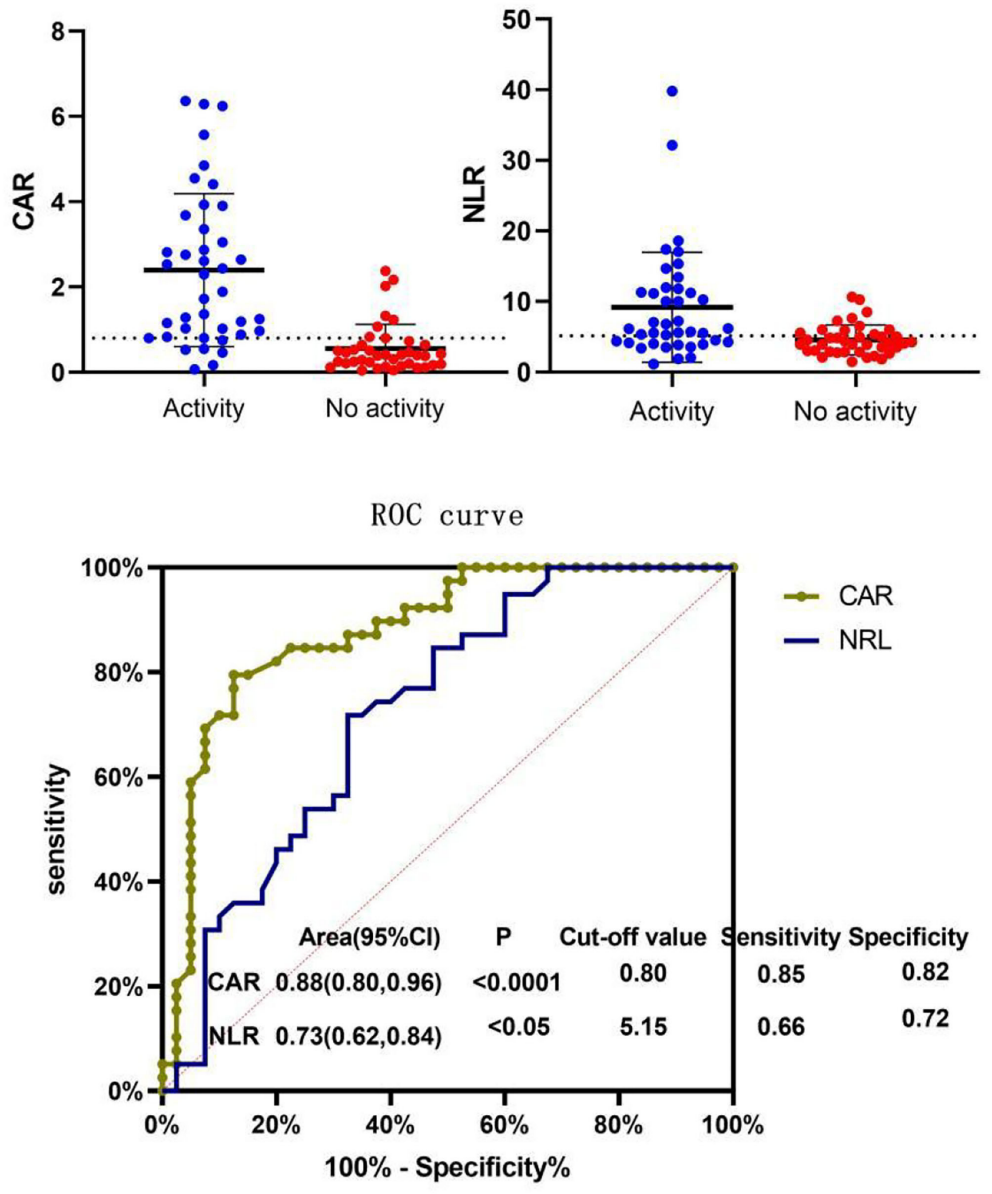
Receiver operating characteristic (ROC) curve of NLR and CAR for BVAS. Patients were divided into the active disease group (BVAS > 15) and the non-active disease group (BVAS ≤ 15) and ROC curve analysis was conducted for the CAR and NLR. The optimal cutoff value for disease activity evaluation using the CAR was 0.80 (sensitivity: 85% and specificity: 82%, *P* < 0.05). The optimal cutoff value for disease activity evaluation using the NLR was 5.15 (sensitivity: 66% and specificity: 72%, *P* < 0.05). CAR, C-reactive protein/albumin ratio; NLR, neutrophil/lymphocyte ratio; MPV, mean platelet volume; BVAS, Birmingham Vasculitis Activity Score.

ROC curves of CAR and NLR relative to BVAS are shown in Figure 3. The optimal cutoff value for maximum specificity and sensitivity of CAR for disease activity prediction was 0.80 and the optimal cutoff value for NLR was 5.15. According to our ROC curve analysis, prediction of disease activity based on the CAR in AAV patients has a sensitivity of 85% and a specificity of 82%, and the sensitivity and specificity of the NLR were 66 and 72%, respectively.

### Relationship Between CAR and NLR and the Cumulative Survival Rate of Newly Diagnosed AAV Patients

**Figure 4:**
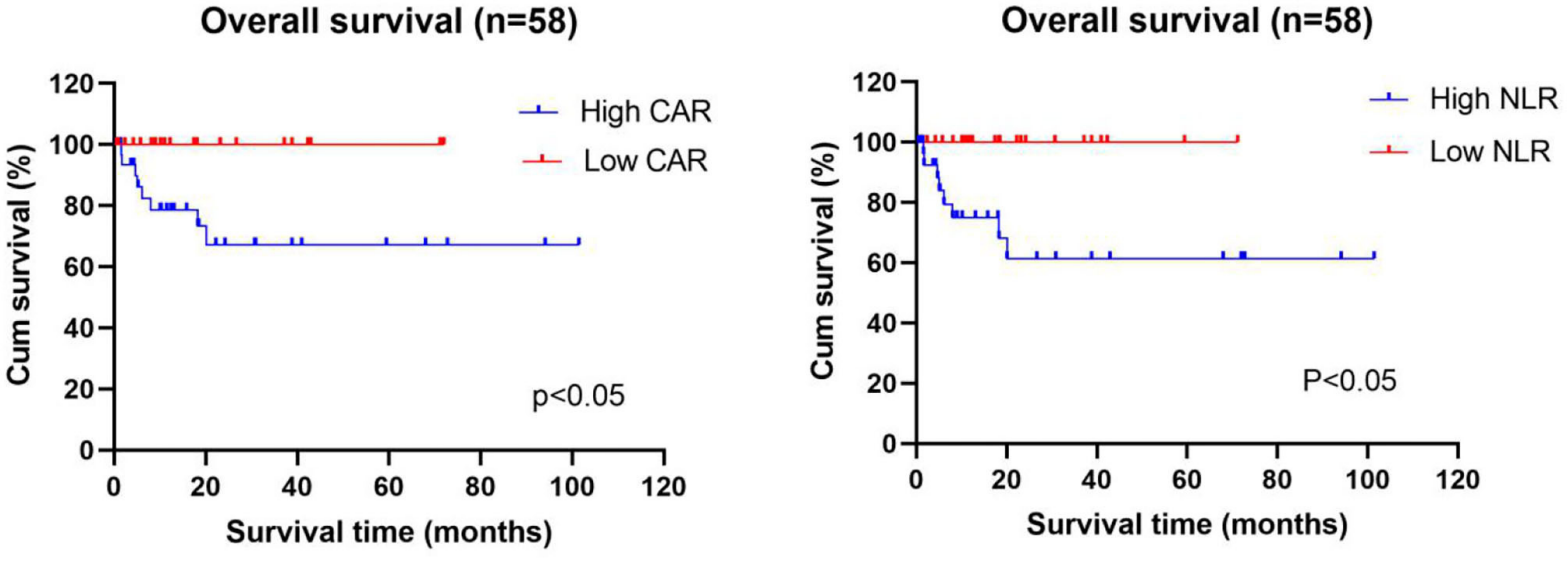
Relationship between the CAR and NLR and the cumulative survival rate of newly diagnosed AAV patients. Kaplan–Meier survival analysis revealed that the all-cause mortality rate was higher in AAV patients with high CAR (≥0.8) and high NLR (≥5.15) than in patients with low CAR (<0.8) and low NLR (<5.15). CAR, C-reactive protein/albumin ratio; NLR, neutrophil/lymphocyte ratio.

The data is current to April 2021 when the searches were last completed and the follow-up was carried out on the diagnosed AAV patients. After excluding 11 patients who were lost to follow-up and 10 patients who did not regularly take medication, 58 patients were included in the survival analysis, of whom 8 died and 50 survived. Patients were divided into the high and low CAR groups and high and low NLR groups based on the optimal cutoff values for CAR and NLR (CAR ≥ 0.8 and NLR ≥ 5.15). As shown in Table 4, lung injury was more common in the high CAR group than in the low CAR group. Indeed, there was markedly different cumulative survival rate between lung injury patients and non-lung injury patients (*P* < 0.05). These data shown that CAR can be used as an effective disease activity marker, and the AAV patients with high CAR might have a poor prognosis. Actually, Kaplan–Meier survival analysis revealed that the all-cause mortality rate was higher in patients with CAR ≥ 0.8 than in patients with CAR < 0.8 (*P* < 0.05). In addition, patients with low NLR also showed a lower cumulative survival rate (*P* < 0.05) (Figure 4).

**Table 4:** Clinical manifestation of high CAR and low CAR, high NLR and low NLR AAV patients.

**Clinical manifestation**	**HighCAR**	**LowCAR**	***P* value**	**High NLR**	**LowNLR**	***P* value**
	**(*n* = 40)**	**(*n* = 39)**		**(*n* = 38)**	**(*n* = 41)**	
General	27	20	0.173	23	24	1.000
Cutaneous	6	1	0.108	3	4	1.000
Mucous membranes/eyes	5	0	0.055	2	3	1.000
Ear nose throat	6	1	0.108	3	4	1.000
Pulmonary	32	11	<0.05	24	19	0.176
Cardiovascular	9	7	0.781	10	6	0.256
Abdominal	2	1	1.000	2	1	0.606
Renal	34	27	0.114	32	29	0.186
Nervous systems	6	4	0.737	5	5	1.000

## Discussion

This study evaluated the relationship between disease activity and CAR, NLR, and MPV in AAV patients. We found that compared with the healthy control group, the CAR, NLR, and MPV levels in newly diagnosed and untreated AAV patients were significantly elevated. With regard to how to quantify disease activity in AAV patients, we selected the BVAS scoring system for quantitative evaluation. This system includes nine sub-systems and the weighted score for new-onset/worsening or persistence of every symptom is different. Therefore, this system is considered to be the most reliable tool for evaluating AAV activity (14). We further found that the BVAS disease activity score of AAV patients was positively correlated with CRP and NLR, and patients with high CAR and high NLR had higher all-cause mortality rates.

CRP is synthesized and degraded in hepatocytes, and serum CRP levels rise drastically within 24–72 h during inflammation. CRP has a short half-life and is usually considered to be the laboratory marker of choice for acute inflammatory diseases (15). Serum ALB is also produced by the liver and severe inflammatory diseases and malnutrition will cause serum ALB levels to decrease (16). In contrast to CRP alone or serum ALB alone, the CAR includes two important inflammation markers with different presentations during inflammation and can maximally reflect disease burden. The assays to detect these two markers are simple and cheap (17–19). A previous study showed that the CAR is related to the all-cause mortality rate in AAV patients (20). The present study found that the CAR in AAV patients was significantly higher than in the healthy control group and showed a significant positive correlation with the BVAS disease activity score. Through plotting of ROC curves, we confirmed that the optimal cutoff value of CAR > 0.80 can be used to predict disease activity. Disease activity significantly affects patient prognosis and organ function. This study also showed that the CAR was related to the all-cause mortality rate in AAV patients.

In the human body, neutrophils account for 50–70% of all circulating leukocytes, are the most abundant circulating leukocytes, and play an important role in innate immunity (21–23). In some autoimmune diseases, neutrophils are considered to be the main source of autoantigens that trigger autoimmune diseases (24, 25). Neutrophil extracellular traps (NETs) secreted by dead neutrophils can capture and kill pathogens, damage endothelial cells, present antigens, promote platelet activation, and participate autoimmune reactions (26–28). The same ANCA can stimulate neutrophils to release NETs that include autoantigens and cause AAV patients to produce autoimmune responses to these components (29). At the same time, lymphocytopenia is considered to be related to inflammation burden, such as in rheumatoid arthritis, Crohn's disease, systemic lupus erythematosus, and vasculitis (30–33). However, some studies found that lymphocytes are negatively correlated with the recurrence rate, with lymphocytopenia associated with a lower recurrence rate (34). NLR is a combination of two independent inflammation markers. Neutrophils are mainly responsible for non-specific and early systemic inflammation while lymphocyte changes occur relatively late and participate in late immune responses. Therefore, a marker that combines two immune cells with different characteristics is more reliable than a single immune cell count and is widely used to evaluate inflammation burden and predict disease prognosis (35, 36). The NLR is related to the severity of many autoimmune diseases, such as psoriasis, rheumatoid arthritis, systemic lupus erythematosus, Behçet's syndrome, and Sjögren syndrome (7, 9–11). The results of this study showed that the NLR was significantly higher in the AAV group than in normal subjects and that it is positively correlated with ANCA disease activity. This shows that the NLR may have clinical value in monitoring ANCA disease activity. By plotting the ROC curve, we determined that the optimal cutoff value for NLR was 5.15. Based on this result, we recommend that more frequent consultation, comprehensive laboratory tests, and evaluation of treatment results may be required in AAV patients with NLR ≥ 5.15.

Platelet count has always been used as a marker of inflammatory disease activity and MPV plays an important role as a deciding factor for the platelet response in many inflammatory diseases (37). MPV can be used to predict severe COVID-19 cases (38) and its elevation is an independent risk factor for coronary artery and peripheral artery diseases (39, 40). In autoimmune diseases, such as Behçet's syndrome, increased MPV can reflect Behçet's syndrome disease activity and predict ocular complications (12). However, the results of some studies also showed that MPV is not associated with mortality and recurrence rates in primary malignant bone tumors (41) and also not associated with COVID-19 severity (42). In a study on systemic lupus erythematosus, both cross-sectional and longitudinal studies found no correlation between disease activity and MPV (43). These contradictory results may be because MPV changes are affected by many factors, such as age, gender, diabetes, obesity, and hypertension (44, 45). Our study found that there is a difference in MPV between AAV patients and healthy controls, but MPV is not associated with the BVAS disease activity score.

Our study showed that the CAR and NLR can be used as two potential markers to reflect the AAV inflammation status, assess disease activity, and predict the chance of survival based on the optimal cutoff values. We recommend that frequent hospital consultation and examinations should be carried out for AAV patients with high CAR and high NLR. Neutrophil count, lymphocyte count, ALB levels, and CRP levels are easily determined and objective markers that can be obtained in almost all medical institutions and facilitate patient and physician evaluation. However, we would like to mention some study limitations. This is a single center study with small sample size and a low number of deaths. At the same time, this is a retrospective study and not all confounding factors can be controlled. For example, medication history, disease history, nutrition status, and comorbidities were not recorded in the patients' medical records. The BVAS combines the clinical characteristics of patients to cross-sectionally evaluate disease activity and has some inter-individual differences and subjectivity. Therefore, the accurate reflection of AAV disease activity is limited. We hope that a future large-sample, multicenter, prospective study can be carried out to validate our results and provide more definite optimal CAR and NLR cutoff values to predict disease activity in AAV patients and promote application in clinical practice.

## Data Availability Statement

The raw data supporting the conclusions of this article will be made available by the authors, without undue reservation.

## Ethics Statement

This study was approved by the Ethics Committee of the Jiangxi Provincial People's Hospital in accordance with the World Medical Association Declaration of Helsinki. The patients/participants provided their written informed consent to participate in this study.

## Author Contributions

YT, NL, HY, and LD reviewed the medical records, analyzed the data, and wrote the first draft. YT, HY, and LD reviewed the literature and finalized the revised manuscript. All authors have read and approved the final manuscript.

## Funding

This work was supported by the National Natural Science Foundation of China (81960296 and 81871286), JiangXi Provincial Natural Science Foundation of China (20192ACB21006), Interdisciplinary Innovation Team, Frontier Science Key Research Project of Jiangxi Provincial People's Hospital (19-008), Long-term (Youth) Project for Leading Innovative Talents in Jiangxi Province (LD), Jiangxi Provincial Clinical Research Center for Rheumatic and Immunologic Diseases (20192BCD42005), Jiangxi Province Medical Leading Discipline Construction Project (Rheumatology), and Provincial and Municipal Joint Construction Projects of Medical Disciplines in Jiangxi Province (Rheumatology).

## Conflict of Interest

The authors declare that the research was conducted in the absence of any commercial or financial relationships that could be construed as a potential conflictof interest.

## Publisher's Note

All claims expressed in this article are solely those of the authors and do not necessarily represent those of their affiliated organizations, or those of the publisher, the editors and the reviewers. Any product that may be evaluated in this article, or claim that may be made by its manufacturer, is not guaranteed or endorsed by the publisher.
