# Corrigendum: Relationship between c-reactive protein/serum albumin ratio, neutrophil/lymphocyte ratio, and ANCA-associated vasculitis activity: A retrospective single center cohort study

**DOI:** 10.3389/fmed.2022.963919

**Published:** 2022-07-25

**Authors:** Yao Tian, Na Liu, Hui Yin, Lihua Duan

**Affiliations:** ^1^Department of Rheumatology and Clinical Immunology, Jiangxi Provincial People's Hospital, Medical College of Nanchang University, Nanchang, China; ^2^Department of Rheumatology and Clinical Immunology, Jiangxi Provincial People's Hospital, The First Affiliated Hospital of Nanchang Medical College, Nanchang, China

**Keywords:** CAR, NLR, associated vasculitis (ANCA), MPV, disease activity

In the published article, there was an error in affiliations 1 and 2. Instead of “^1^ Department of Rheumatology and Clinical Immunology, Jiangxi Provincial People's Hospital, The First Affiliated Hospital of Nanchang Medical College, Nanchang, China,” it should be “^1^ Department of Rheumatology and Clinical Immunology, Jiangxi Provincial People's Hospital, Medical College of Nanchang University, Nanchang, China.” Instead of “^2^ Department of Rheumatology and Clinical Immunology, Jiangxi Provincial People's Hospital, Nanchang University, Nanchang, China,” it should be “^2^ Department of Rheumatology and Clinical Immunology, Jiangxi Provincial People's Hospital, The First Affiliated Hospital of Nanchang Medical College, Nanchang, China.”

The authors apologize for this error and state that this does not change the scientific conclusions of the article in any way. The original article has been updated.

## Publisher's note

All claims expressed in this article are solely those of the authors and do not necessarily represent those of their affiliated organizations, or those of the publisher, the editors and the reviewers. Any product that may be evaluated in this article, or claim that may be made by its manufacturer, is not guaranteed or endorsed by the publisher.

